# Zeb1 for RCP-induced oral cancer cell invasion and its suppression by resveratrol

**DOI:** 10.1038/s12276-020-0474-1

**Published:** 2020-07-30

**Authors:** Jin Young Kim, Kyung Hwa Cho, Bo Young Jeong, Chang Gyo Park, Hoi Young Lee

**Affiliations:** Department of Pharmacology, College of Medicine, Konyang University, Daejeon, Republic of Korea

**Keywords:** Oral cancer, Cell invasion

## Abstract

Rab coupling protein (RCP) is upregulated in head and neck squamous cell carcinoma (HNSCC) and is correlated with the progression and survival of patients. However, the role of RCP in one of the aggressive types of HNSCC, oral squamous cell carcinoma (OSCC), remains elusive. In the present study, we identified the important role of Zeb1 in RCP-induced OSCC epithelial-to-mesenchymal transition (EMT) and invasion. RCP induces Zeb1 expression, and silencing Zeb1 expression significantly inhibits RCP-induced OSCC invasion. In addition, Zeb1 upregulates MT1-MMP expression to promote OSCC EMT and invasion. Furthermore, we observed that the β1 integrin/EGFR/β-catenin signaling cascade mediates RCP-induced Zeb1 expression to promote OSCC invasion. Notably, we provide evidence that resveratrol (REV) strongly inhibits RCP-induced Zeb1 expression through blocking β1 integrin endosome recycling and EGFR activation, leading to suppression of RCP-induced OSCC invasion, demonstrating the important role of RCP in OSCC invasion and its reversion by REV. Collectively, the present study provides evidence for the first time that RCP aggravates OSCC invasion through increasing Zeb1 expression and subsequently upregulating MT1-MMP expression and that this process is reversed by REV, providing novel biomarkers and indicating the therapeutic potential of REV in OSCC.

## Oral cancer: Stopping the spread

Resveratrol, a naturally occurring compound present in grape skins, peanuts, and blueberries, reduces the ability of oral squamous cell carcinoma (OSCC) cells to spread to other parts of the body. Over half a million new cases of OSCC are diagnosed each year, and spread beyond the mouth is the most common cause of death. Hoi Young Lee at Konyang University, Daejon, Republic of Korea, and co-workers investigated whether a protein called RCP, which increases the invasiveness of many but not all types of cancer, is implicated in aggressive spread of OSCC. They found that RCP does increase the invasiveness of OSCC. Resveratrol is known to suppress the spread of many cancers, and strongly curtailed the spread of OSCC by blocking RCP activity. These results shed light on OSCC invasiveness, and offer a potential new treatment.

## Introduction

Head and neck squamous cell carcinoma (HNSCC) stems from the epithelia of the aerodigestive tract, including the oral cavity^1^. The prevalence of HNSCC is increasing worldwide, with nearly 800,000 new diagnoses and 350,000 cancer-associated deaths annually^1–3^. Similar to other types of cancer, metastasis to distant organs is the most common cause of death in HNSCC patients. Metastasis is a complex multistep process that includes epithelial-mesenchymal transition (EMT), which is highlighted by reduced expression of E-cadherin through upregulation of EMT-activating transcription factors (EMT-TFs), such as Zeb proteins^4,5^.

Identified as a Rab4 and Rab11 effector protein, Rab coupling protein (RCP) originates from the *RAB11FIP1* gene and is associated with the proliferation and progression of various cancers^6,7^. In particular, RCP is overexpressed in numerous types of cancer, including HNSCC, and augments cancer invasion and metastasis^8–11^. Mechanistically, RCP promotes cancer cell EMT and metastasis by protecting β1 integrin from lysosomal digestion and activating EGFR^6,11^. In addition, RCP participates in EphA2 trafficking during cell repulsion^12^ and mediates mutant p53-induced cancer invasiveness^13,14^. However, RCP has also been suggested as an important negative regulator in Her-2-positive breast cancer through the lysosomal degradation of Her-2^15^.

In the present study, we investigated the effect of RCP on one of the aggressive types of HNSCC, oral squamous cell carcinoma (OSCC)^16^, and identified that RCP induces OSCC EMT and invasion through Zeb1 and MT1-MMP expression. More interestingly, we revealed that the natural phytoalexin resveratrol (REV) efficiently attenuates RCP-induced OSCC invasion through the downregulation of β1 integrin and Zeb1 expression, providing novel biomarkers and potential therapeutic options in OSCC.

## Materials and methods

### Cell culture and reagents

OSCC cell lines YD-9 and YD-38 were obtained from the Korean Cell Line Bank (Seoul, Korea). YD-10B cells were a gift from Dr. J.I. Yook (Yonsei University College of Dentistry, Seoul, Korea). Oral cancer-associated fibroblasts (CAFs) were generously provided by Dr. X.L. Zhang (Yonsei University College of Dentistry, Seoul, Korea). Oral CAFs were cultured in DMEM and F-12 Ham mixed in a 3:1 ratio and supplemented with 10% FBS and 1% penicillin/streptomycin. All OSCC cells were cultured in RPMI supplemented with 10% FBS and 1% penicillin/streptomycin at 37 °C under 5% CO_2_ in a humidified incubator. Gefitinib was purchased from Selleckchem (Houston, TX). REV was purchased from Sigma-Aldrich (St Louis, MO). All other reagents were of the purest grade available.

### siRNA and plasmid transfection

Cells were transiently transfected using Lipofectamine 3000 or RNAiMAX (Invitrogen, Carlsbad, CA) as described previously^11^. RCP and Zeb1 constructs were in the pEGFP-C3 and pCMV6A vectors, respectively. β1 integrin cDNA was added to the pcDNA3 vector. Each empty vector was used as the negative control. RCP and β1 integrin constructs were provided by Jim Norman (Beaston Cancer Institute, Glasgow, UK) and Dr. Y.S. Lee (Ewha Woman’s University, Seoul, Korea), respectively. The vector for Zeb1 was purchased from Switchgear Genomics (Carlsbad, CA). siRNAs against RCP, Zeb1, N-cadherin, and β1 integrin were purchased from Sigma-Aldrich, and β-catenin siRNA was purchased from Santa Cruz Biotechnology (Dallas, Texas). Control scrambled siRNA was purchased from Invitrogen. Lentivirus packaging and transduction were performed with a Second Generation packaging mix kit (Abm, Richmond, BC, Canada) according to the manufacturer’s protocol. Briefly, the recombinant lentivirus was produced by transfecting HEK293T cells with expression and packaging vectors. The supernatant medium was harvested twice at 24 h and 48 h. The prepared YD-10B cells were transduced with the virus-containing supernatant with polybrene (5 mg/ml, Merck Millipore, Darmstadt, Germany). The permanent RCP transfectants were selected using puromycin (1 μg/ml, Thermo Fisher Scientific Inc., Rockford, IL) for 7 days, and the expression of RCP was evaluated by immunoblotting.

### Quantitative RT-PCR

Isolation of total cellular RNA and subsequent analysis were performed as previously described^11,17^. Complementary DNA was amplified using an iQ5 Real-Time PCR Detection System (Bio-Rad Laboratories, Hercules, CA) with the following primer sets: RCP, 5′-GGATGTCTCCGAATCTTCCA-3′ (forward) and 5′-CCGTCATCAGAGACAGCAAA-3′ (reverse); MT1-MMP, 5′-TTGGACTGTCAGGAATGAGG-3′ (forward) and 5′-GCAGCACAAAATTCTCCGTG-3′ (reverse); MMP2, 5′-ATGACAGCTGCACCACTGAG-3′ (forward) and 5′-AGTTCCCACCAACAGTGGAC-3′ (reverse); MMP9, 5′-GTGCCATGTAAATCCCCACT-3′ (forward) and 5′-CTCCACTCCTCCCTTTCCTC-3′ (reverse); uPA, 5′-GTGGCCAAAAGACTCTGAGG-3′ (forward) and 5′-GCCGTACATGAAGCAGTGTG-3′ (reverse); Zeb1, 5′-AAGAATTCACAGTGGAGAGAAGCCA-3′ (forward) and 5′-CGTTTCTTGCAGTTTGGGCATT-3′ (reverse); and glyceraldehyde-3-phosphate dehydrogenase (GAPDH), 5′-A CAGTCAGCCGCATCTTCTT-3′ (forward) and 5′-ACGACCAAATCCGTTGACTC-3′ (reverse). The GAPDH gene was used as a control for calculating dCt. The real-time PCR data were analyzed using the 2−(ddCt) method.

### Immunoblotting

Immunoblotting was analyzed as previously described^18^. Briefly, the cell lysates were resolved by SDS-PAGE. PVDF membranes with proteins were blocked and incubated for 30 min. Antibodies against RCP (1:1000, 12849S), Snail (1:1000, 3879S), N-cadherin (1:1000, 4061S), Zeb1 (1:1000, 3396S), p-EGFR (1:1000, 4407S), EGFR (1:1000, 2232 S), MT1-MMP (1:1000, 13130S) and nonphospho (NP) β-catenin (1:1000, 19807S) were purchased from Cell Signaling Inc. (Danvers, MA). Antibodies against GAPDH (1:3000, sc-47724), Twist (1:1000, sc-15393), β-catenin (1:1000, sc-7963), and β1 integrin (1:1000, sc-53711) were purchased from Santa Cruz Biotechnology. An antibody against E-cadherin (1:1000, 610182) was obtained from BD Biosciences (Franklin Lakes, NJ). The immunoreactive bands were exposed via ECL (Thermo Fisher Scientific Inc.) using an Amersham Imager 600 (GE Healthcare, Buckinghamshire, UK).

### Immunofluorescence

Immunofluorescence was performed as previously described^19^. Briefly, immunofluorescence detection was conducted with antibodies against RCP (1:500, Cell Signaling Technology), E-cadherin (1:500, Santa Cruz Biotechnology), β1 integrin (1:500, Santa Cruz Biotechnology), MT1-MMP (1:500, Santa Cruz Biotechnology), and LAMP1 (1:500, Abcam, Cambridge, UK) overnight. The cells were washed with ice-cold PBS and incubated with Cy3-conjugated goat anti-rabbit IgG (1:500, Jackson ImmunoResearch, PA), Cy2-conjugated goat anti-mouse IgG (1:500, Jackson ImmunoResearch) and Cy5-conjugated goat anti-rat IgG (1:500, Jackson ImmunoResearch). The nuclei of cells were marked with 4′,6′-diamidino-2-phenylindole (DAPI, Invitrogen). The cells were observed by confocal microscopy (x600, LSM710, Carl Zeiss).

### In vitro invasion and wound healing assay

Invasion and wound healing analyses were performed as previously described^11,20^. Briefly, 0.4 × 10^6^–1 × 10^6^ cells were loaded in the upper well of the invasion chamber. After incubation for 13–15 h at 37 °C, the cells that invaded the membrane were fixed and stained with Diff-Quik reagents (Sysmex Co., Kobe, Japan). The invaded cells were calculated by counting the number of cells in three arbitrary high-power fields for each replicate (×200) with a light microscope. The results were derived from three independent experiments. For wound healing analysis, the cells were seeded in 6-well plates for 24 h and then transfected with the indicated vector and siRNA. After serum starvation, the cells were scraped with a 200 μl pipette tip. The gap distance of cells was observed after 24 h incubation in the same locations.

### Three-dimensional (3D) Matrigel culture

Three-dimensional (3D) Matrigel culture was executed as previously described^11^. Briefly, cells were suspended in 2% Matrigel and put over a layer of polymerized 100% Matrigel at 1 × 10^4^ cells/ml in an eight-well chamber slide (Nunc, Littleton, CO). RPMI culture medium was replaced once every two days. Cultures were analyzed after 7 days of cultivation.

### Three-dimensional (3D) gel invasion assay

3D gel invasion was analyzed as described previously with some modifications^21–23^. Cells were embedded in a mixture of 20% type I collagen (Nitta Gelatin Inc, Cellmatrix Type I-P, Japan) and Matrigel (BD Biosciences) in Transwell (0.3 μm pore size, Corning, Acton, MA). Oral CAFs and YD-10B cells were labeled with DiI (Oral CAFs; Thermo Fisher Scientific, Waltham, MA) and DiO (YD-10B cells; Thermo Fisher Scientific), respectively. Oral CAFs (2 × 10^4^) and YD-10B cells (2 × 10^4^) were mixed in 200 μl of medium (1:1 mixture of RPMI and medium of oral CAFs) supplemented with 0.2% FBS and plated on gels. The low chamber of the Transwell was filled with 800 μl of the above medium with 10% FBS. After 5 days, the embedded gel was sectioned without a fixture, and the cells were analyzed by fluorescence confocal microscopy. In these images, the distance of invaded cells was measured from eight different positions and calculated by the ZEN blue edition program of Carl Zeiss Microscopy GmbH. The distance in μm was calculated as described previously^24^.

### Cell viability

The cell viability assay was determined as previously described^20,25^. Briefly, cells were seeded in 96-well plates, serum-starved and treated with or without REV for 24 h. The absorbance was calculated at 540 nm using a SYNERGY/HTX ELISA plate reader (BioTek, Winooski, VT).

### Proteome extraction assay

Proteome extraction assays were performed according to the manufacturer’s protocol (Merck-539790). Briefly, YD-10B cells were transfected with the indicated vectors for 72 h. After washing with cold washing buffer, the cells were added to fraction buffers to isolate supernatants with proteins in the cytosol (buffer I), membrane (buffer II) and nucleus (buffer III). Supernatants from buffers I and III were resolved by SDS-PAGE.

### Statistical analysis

Data are shown as the means ± standard deviation (SD). Differences between two groups were assessed with SigmaPlot software (Systat Software, San Jose, CA) using Student’s *t*-test, and statistical significance was set at *p*-values less than 0.05. Differences among three or more groups were estimated by analysis of variance followed by Bonferroni multiple comparison tests.

## Results

### RCP induces OSCC EMT and invasiveness

**Fig. 1:**
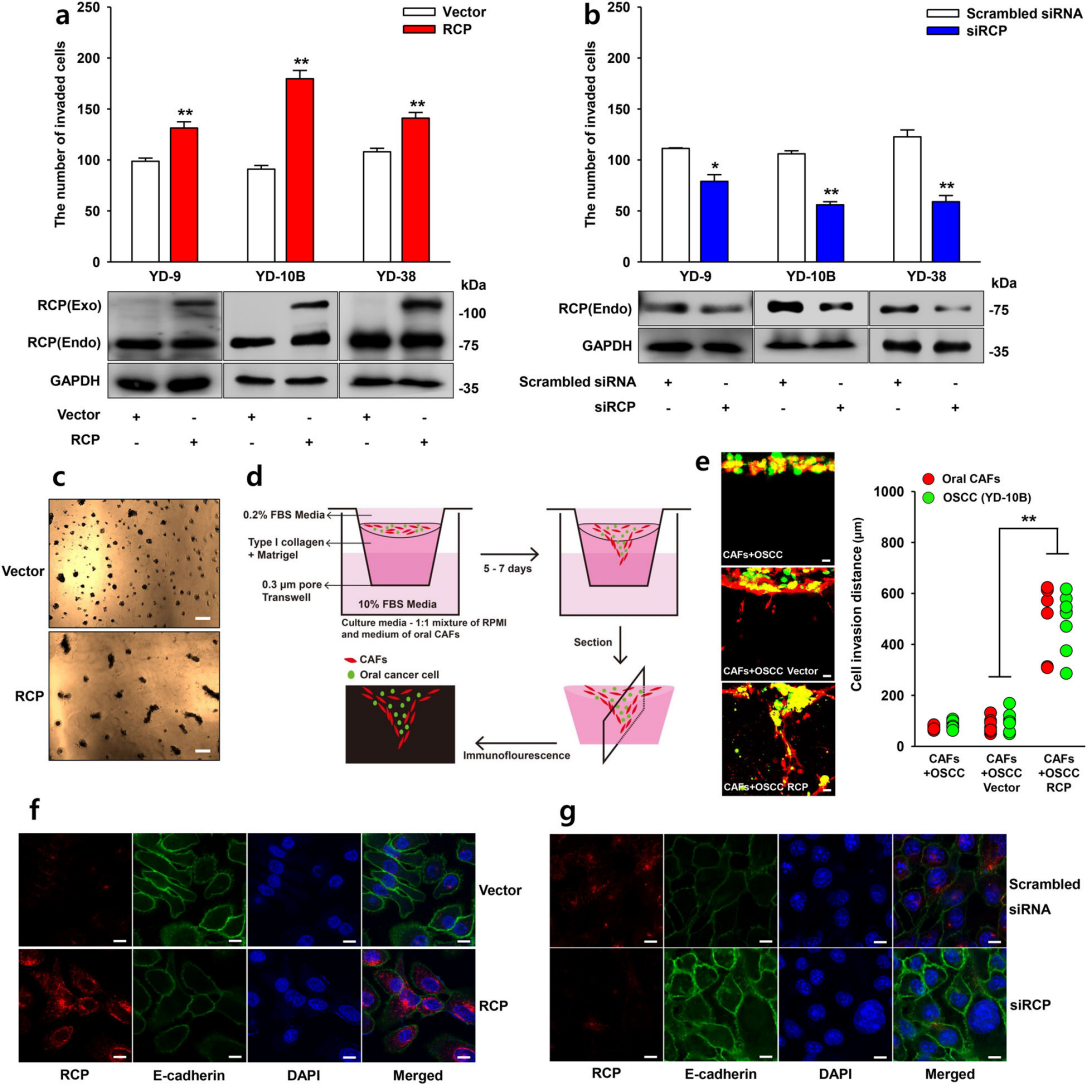
RCP induces OSCC EMT and invasiveness. **a**, **b** OSCC cells were transfected with the indicated vectors or siRNAs. Cell invasion assays were performed with media without any attractant as the control (error bars, ±SD: ***p < 0.001 and **p < 0.01 vs control). **c** 3D Matrigel culture showing the growth of transfected YD-10B cells with vector or RCP. Image original magnification ×40, scale bar, 200 μm. **d** Schematic drawing showing the coculture of CAFs and OSCC cells in the 3D Matrigel invasion system. YD-10B cells were transfected with vector or RCP. CAFs, and YD-10B cells were labeled with staining dye (CAFs: red, YD-10B: green) and then incubated for 7 days for 3D gel invasion analysis. The gel was sectioned without fixation, and then the cells were analyzed by fluorescence confocal microscopy. **e** Compared to the vector control, RCP overexpression enhanced OSCC cell invasive potential when cocultured with oral CAFs. In these images, the distance of invaded cells was measured in eight different positions and calculated by the ZEN blue edition program of Carl Zeiss Microscopy GmbH. Image original magnification, ×50, scale bar, 50 μm. **f**, **g** The cells were transfected with the indicated vectors or siRNAs, and the expression of E-cadherin was visualized by florescence confocal microscopy. Image original magnification, ×600, scale bar, 10 μm. The results shown are representative of three experiments with similar results and indicate the mean ± SD of three experiments.

To determine the role of RCP in OSCC invasiveness, we first determined the level of RCP expression in OSCC. We observed that all tested OSCCs express detectable amounts of RCP (Supplementary Fig. 1). In addition, ectopic expression of RCP significantly increased their invasiveness (Fig. 1a). By contrast, silencing of RCP expression markedly attenuated the invasiveness of OSCC cells (Fig. 1b). Moreover, we observed the increased growth of YD-10B cells on 3D Matrigel when they were transfected with RCP (Fig. 1c). To examine the effects of RCP on coordinated invasion by oral CAFs and cancer cells, a 3D gel invasion assay was utilized, which reflects cancer invasion in vivo. Cells were labeled with distinguishable fluorescent dyes and placed on top of a gel containing type I collagen and Matrigel (Fig. 1d). Interestingly, compared to the vector control, overexpression of RCP resulted in a higher invasive potential in cells co-cultured with oral cancer-associated fibroblasts (CAFs) in 3D Matrigel (Fig. 1e), indicating that RCP reorganizes the microenvironment to augment the invasiveness of OSCC. Furthermore, immunofluorescence analysis showed that RCP induces EMT in OSCC. RCP efficiently reduced the E-cadherin expression of YD-10B cells, while transfection of the cells with RCP siRNA dramatically increased E-cadherin expression in YD-38 cells (Fig. 1f, g). Therefore, these data imply the critical role of RCP in OSCC EMT and invasion.

### Zeb1 is important for RCP-induced OSCC invasion

**Fig. 2:**
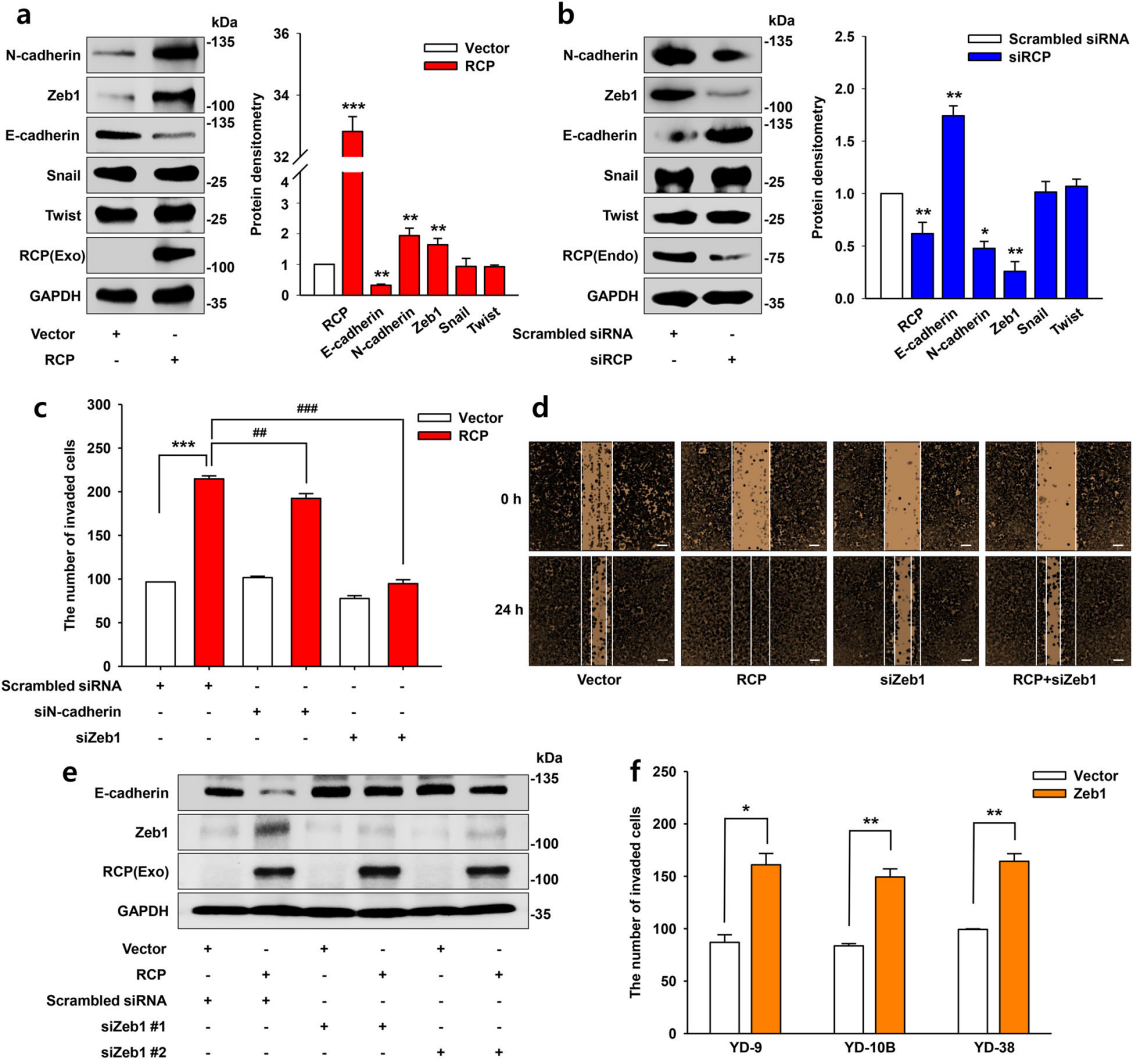
Zeb1 is important for RCP-induced OSCC invasion. **a**, **b** The cells were transfected with the indicated vectors or siRNAs. The expression of EMT-TFs was measured by immunoblotting. The bands were quantified by ImageJ densitometric analysis and normalized to the control vector bands (error bars, ±SD: ***p < 0.001, **p < 0.01 and *p < 0.05 *vs* control vector). **c** YD-10B cells were transfected with the indicated vectors and siRNAs. Cell invasion assays were performed with media without any attractant as the control (error bars, ±SD: **p < 0.01 *vs* control vector, ^##^*p* < 0.01 and ^###^*p* < 0.001 *vs* RCP). **d** YD-10B cells were transfected with the indicated vectors and siRNAs. Wound healing was performed. Image original magnification x40, scale bar, 200 μm. **e** YD-10B cells were transfected with the indicated vectors and siRNAs. E-cadherin expression was assessed by immunoblotting. **f** The cells were transfected with or without Zeb1. Cell invasion assays were performed (error bars, ±SD: **p < 0.01 and *p < 0.05 *vs* control vector). The results shown are representative of three experiments with similar results and indicate the mean ± SD of three experiments.

We next determined whether modulation of the expression level of RCP alters the expression of EMT-TFs in OSCC. Ectopic expression of RCP in YD-10B cells prominently increased N-cadherin and Zeb1 expression (Fig. 2a). In contrast, silencing of RCP expression in YD-38 cells reduced and induced the expression of these EMT-TFs and E-cadherin, respectively (Fig. 2b). To determine the roles of these EMT-TFs in RCP-induced OSCC invasion, we transfected YD-10B cells with siRNAs targeting N-cadherin and Zeb1 and observed that silencing Zeb1 expression shows more dramatic inhibition of RCP-induced YD-10B cell invasion than silencing N-cadherin (Fig. 2c). Likewise, Zeb1 siRNA markedly inhibited RCP-induced cancer cell migration (Fig. 2d). In addition, siRNAs targeting Zeb1 attenuated the reduction in E-cadherin expression induced by RCP (Fig. 2e). Furthermore, ectopic expression of Zeb1-induced OSCC invasion (Fig. 2f), indicating that Zeb1 is sufficient to increase OSCC invasion. Therefore, these data provide evidence that Zeb1 is associated with RCP-induced OSCC EMT and invasion.

### RCP induces Zeb1 expression through the β1 integrin/EGFR signaling axis

**Fig. 3:**
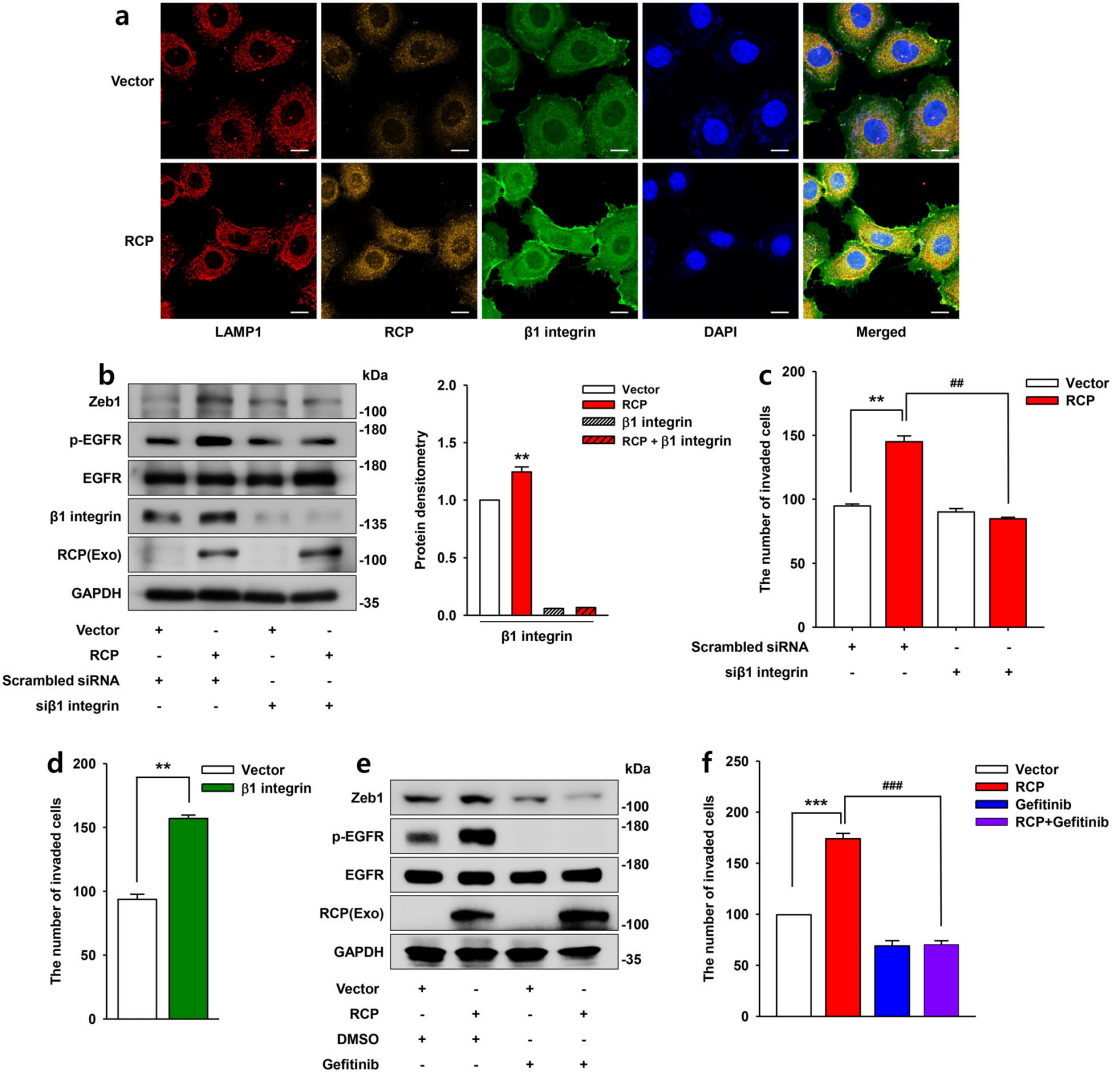
RCP induces Zeb1 expression through the β1 integrin/EGFR signaling axis. **a** The expression of β1 integrin was visualized by immunofluorescence. Image original magnification, ×600, scale bar, 10 μm. **b** YD-10B cells were transfected with the indicated vectors and siRNAs. Immunoblotting. **c**, **d** YD-10B cells were transfected with the indicated vectors and siRNAs. Cell invasion assays were performed (error bars, ±SD: **p < 0.01 vs control vector, ^##^*p* < 0.01 vs RCP). **e** YD-10B cells were sequentially transfected with or without RCP, serum-starved and treated with gefitinib (1 μM) for 24 h. Immunoblotting. **f** YD-10B cells were transfected with RCP followed by pretreatment with gefitinib (1 μM) for 1 h. Cell invasion assays were performed (error bars, ±SD: ***p < 0.001 vs control vector, ^###^*p* < 0.01 vs RCP). The results shown are representative of three experiments with similar results and indicate the mean ± SD of three experiments.

Since RCP has been known to exert its oncogenic property by recycling β1 integrin and thereby EGFR activation^6^, we compared the level of expression and the location of β1 integrin in the cells overexpressing RCP and the control. Immunofluorescence data showed that RCP markedly upregulates the level of β1 integrin expression in the plasma membrane of OSCC compared to the control. RCP was mainly detected in the cytosol. Congruently, we observed increased microspikes stained with β1 integrin at the plasma membrane by RCP (Fig. 3a). Furthermore, immunoblotting analysis revealed increased levels of β1 integrin expression and phospho-EGFR by RCP. However, silencing β1 integrin expression dramatically decreased RCP-induced Zeb1 expression (Fig. 3b) as well as OSCC invasion (Fig. 3c). In addition, overexpression of β1 integrin-induced OSCC invasion (Fig. 3d). Moreover, treatment of the cells with a specific pharmacological inhibitor of EGFR, gefitinib, abolished RCP-induced Zeb1 expression (Fig. 3e) and OSCC invasion (Fig. 3f). Therefore, these data suggest that Zeb1 is critical for RCP-induced OSCC invasion through β1 integrin and EGFR activation.

### The β-catenin/TCF4 complex mediates RCP-induced Zeb1 expression

**Fig. 4:**
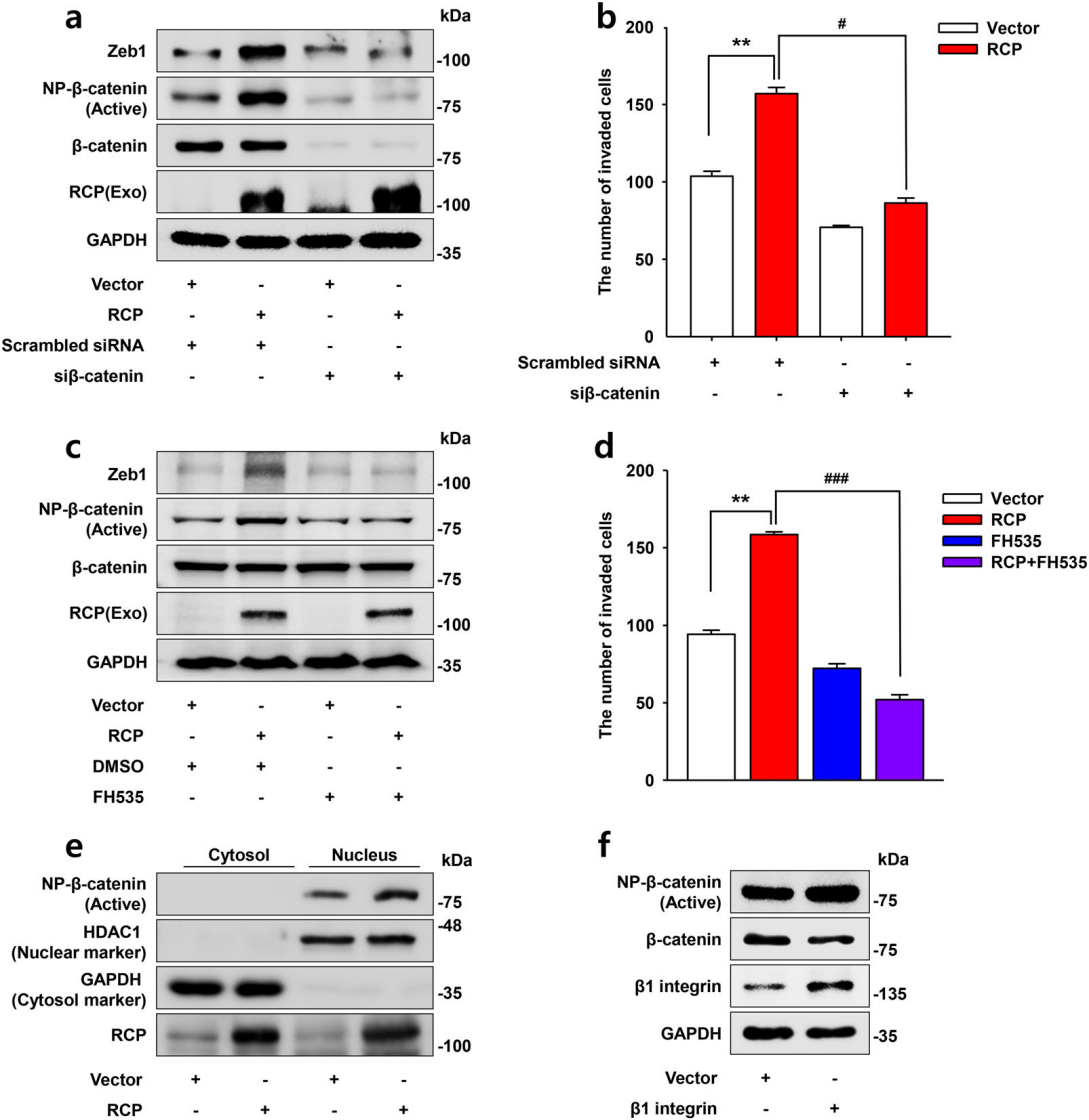
The β-catenin/TCF4 complex mediates RCP-induced Zeb1 expression. **a** YD-10B cells were transfected with the indicated vectors and siRNAs. Immunoblotting. **b** YD-10B cells were transfected with the indicated vectors and siRNAs. Cell invasion assays were performed against media without any attractant (error bars, ±SD: **p < 0.01 vs control vector, ^#^*p* < 0.05 vs RCP). **c** YD-10B cells were sequentially transfected with RCP, serum-starved and treated with FH535 for 24 h. Immunoblotting. **d** YD-10B cells were transfected with RCP followed by pretreatment with FH535 for 1 h. Cell invasion assays were performed against media without any attractant (error bars, ±SD: **p < 0.01 vs control vector, ^###^*p* < 0.001 vs RCP). **e** YD-10B cells were transfected with or without RCP. NP β-catenin expression was assessed by proteome extraction assay. HDAC1 and GAPDH were used as nuclear markers and cytosol markers, respectively. **f** YD-10B cells were transfected with or without β1 integrin. NP-β-catenin expression was assessed by immunoblotting. The results shown are representative of three experiments with similar results and indicate the mean ± SD of three experiments.

Given that β-catenin with TCF4 binds to the Zeb1 promoter and induces its expression as well as tumor progression^26^, we next explored whether β-catenin is implicated in Zeb1 expression in OSCC. RCP markedly increased active β-catenin expression (Fig. 4a). However, silencing of β-catenin expression or incubation of the cells with a selective pharmacological inhibitor of β-catenin, FH535, profoundly reduced RCP-induced Zeb1 expression (Fig. 4a, c) and invasion (Fig. 4b, d). In addition, we noticed that RCP increases the level of active β-catenin expression in the nucleus (Fig. 4e). Interestingly, overexpression of β1 integrin also upregulated active β-catenin expression (Fig. 4f), confirming that RCP, and thereby β1 integrin, induce translocation of β-catenin to upregulate Zeb1 expression. Therefore, these data indicate that β-catenin located downstream of β1 integrin is implicated in RCP-induced Zeb1 expression.

### RCP induces MT1-MMP for OSCC invasion

**Fig. 5:**
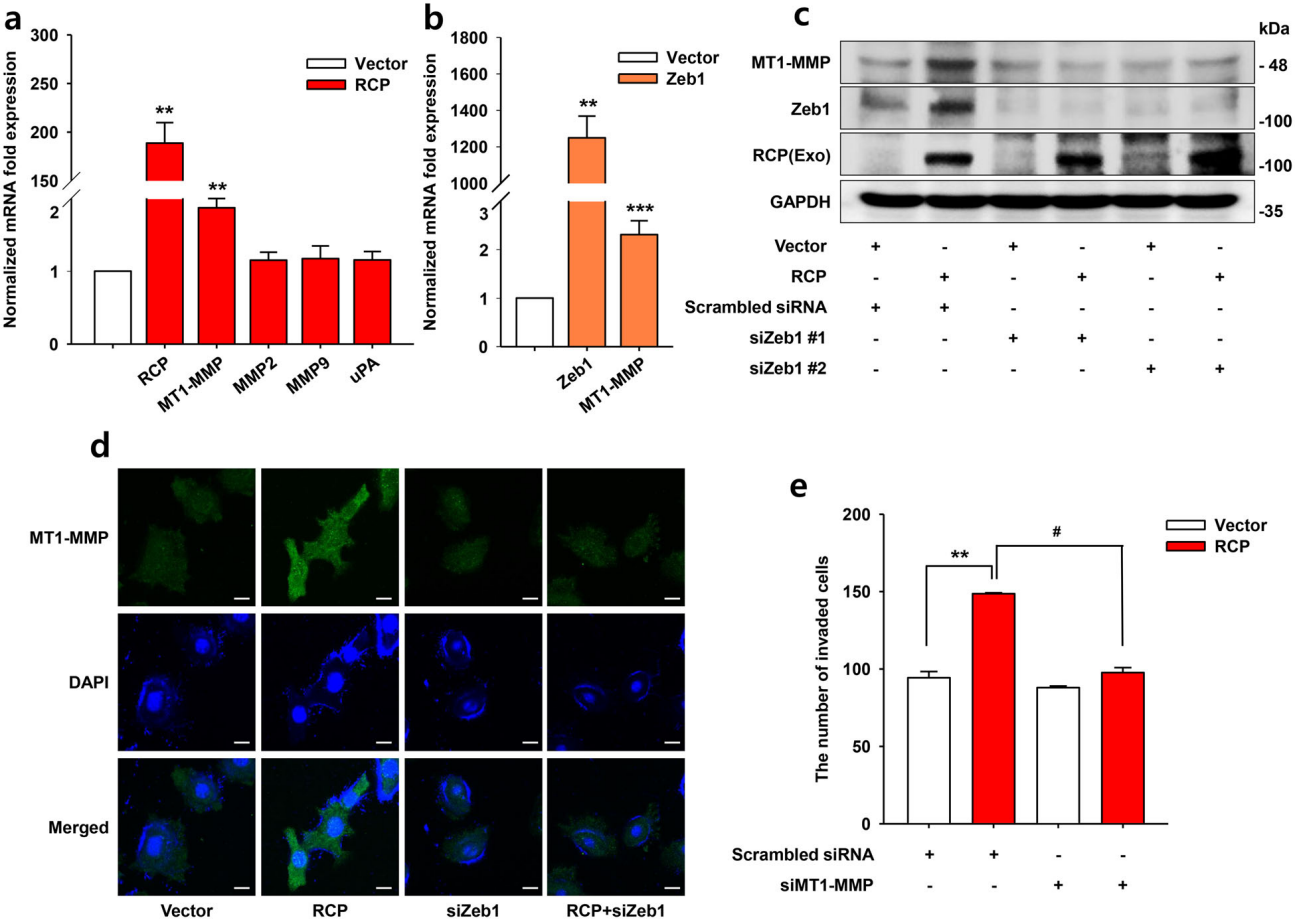
RCP induces MT1-MMP to promote OSCC invasion. **a**, **b** YD-10B cells were transfected with or without (**a**) RCP and (**b**) Zeb1. qRT-PCR analysis of matrix metalloproteinases (MMP2, MMP9, MT1-MMP) and urokinase-type plasminogen activator (uPA) mRNA (error bars, ±SD: ***p < 0.001 and **p < 0.01 vs control vector). **c** YD-10B cells were transfected with the indicated vectors and siRNAs. Immunoblotting. **d** The expression of MT1-MMP was visualized by immunofluorescence. Image original magnification, ×600, scale bar, 10 μm. **e** YD-10B cells transfected with the indicated vectors and siRNAs. Cell invasion assays were performed with media without any attractant as the control (error bars, ±SD: **p < 0.01 vs control vector, ^#^*p* < 0.05 vs RCP). The results shown are representative of three experiments with similar results and indicate the mean ± SD of three experiments.

To specify a critical factor for RCP-induced and thereby Zeb1-induced OSCC invasion, we determined whether RCP modulates the transcript expression of several important proteolytic enzymes for OSCC invasiveness. RCP increased only the MT1-MMP transcript among the tested factors (Fig. 5a). Consistent with these results, the ectopic expression of Zeb1-induced MT1-MMP transcript expression (Fig. 5b). In addition, silencing Zeb1 expression significantly inhibited RCP-induced MT1-MMP expression (Fig. 5c, d), suggesting that Zeb1 mediates MT1-MMP expression in OSCC. Notably, silencing of MT1-MMP expression ablated RCP-induced OSCC invasion (Fig. 5e). Therefore, these results indicate that MT1-MMP is located downstream of Zeb1 and is necessary for RCP-induced OSCC invasion.

### REV mitigates RCP-induced OSCC invasion

**Fig. 6:**
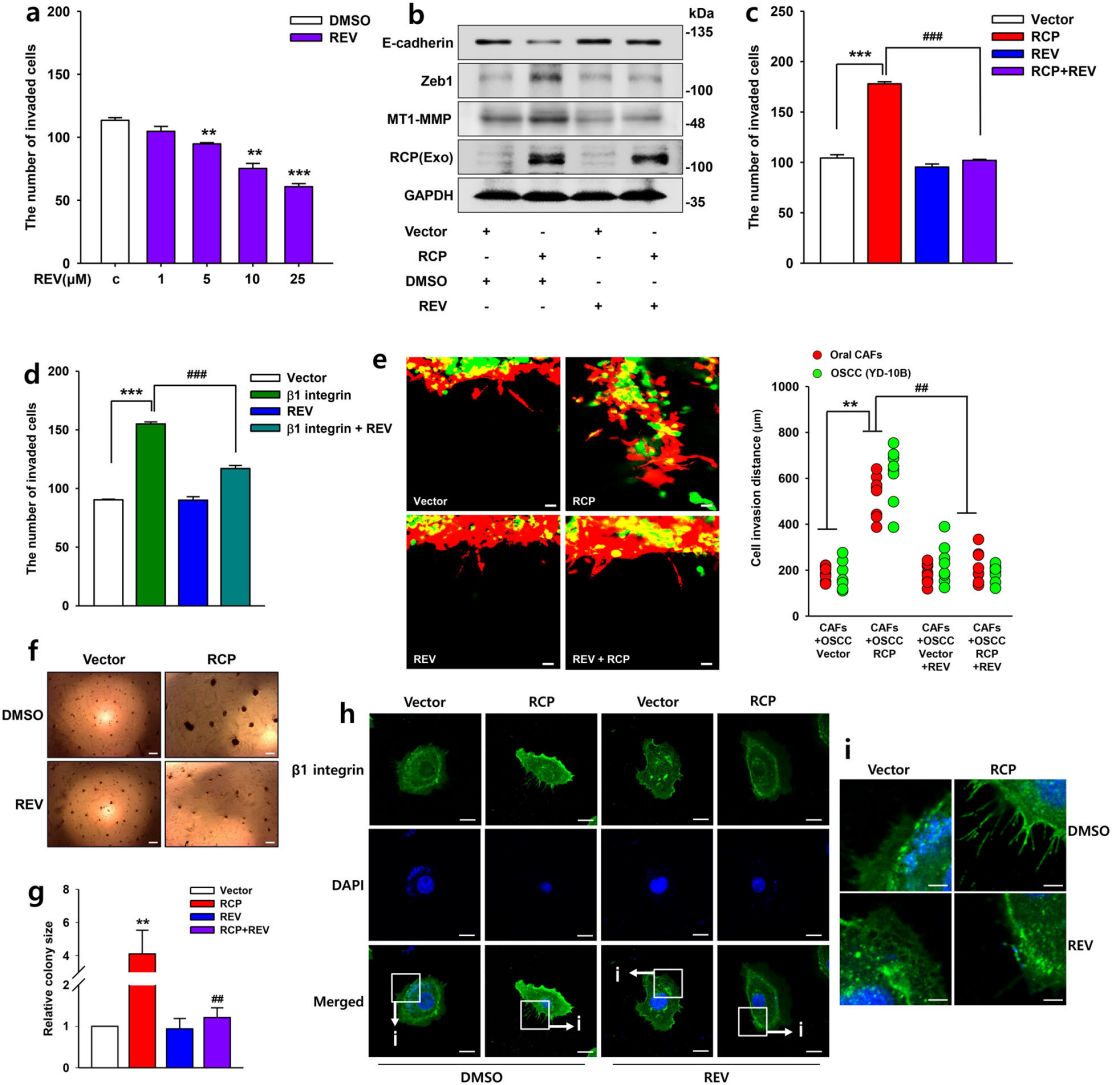
REV mitigates RCP-induced OSCC invasion. **a** YD-10B cells were treated with the indicated amounts of REV for 24 h. Cell invasion assays were performed (error bars, ±SD: ***p < 0.001 and **p < 0.01 vs control vector). **b** YD-10B cells were sequentially transfected with RCP, serum-starved and treated with REV (25 μM) for 24 h. Immunoblotting. **c**, **d** YD-10B cells were transfected with or without (**c**) RCP and (**d**) β1 integrin followed by pretreatment with REV (25 μM) for 1 h. Cell invasion assays were performed against media without attractant (error bars, ±SD: ***p < 0.001 vs control vector, ^###^*p* < 0.001 *vs* RCP). **e** YD-10B cells were transfected with or without RCP, followed by treatment with REV (25 μM). CAFs and YD-10B cells were labeled with staining dye (CAFs: red, YD-10B: green) followed by incubation for 7 days for 3D gel invasion analysis. In these images, the distance of invaded cells was measured in eight different positions and calculated by using the ZEN blue edition program of Carl Zeiss Microscopy GmbH. Image original magnification, ×50, scale bar, 50 μm. **f**, **g** 3D Matrigel culture showing the growth of stably transfected YD-10B cells with or without RCP. Image original magnification, ×40, scale bar, 200 μm. Cell colonies were quantified by ImageJ densitometric analysis and normalized to the control colony. **h**, **i** The expression of β1 integrin was visualized by immunofluorescence. The image in (**i**) is a ×4 magnification of the white box in (**h**). Image original magnification, scale bar, 10 μm (**h**); scale bars, 5 μm (**i**). The results shown are representative of three experiments with similar results and indicate the mean ± SD of three experiments.

Since REV has strong anti-metastatic effects on various cancer cells^27^, we assessed whether REV attenuates RCP-induced OSCC invasion. YD-10B cells had survival rates of 85% or more with REV concentrations up to 25 μM (Supplementary Fig. 2), indicating that REV at these concentrations does not cause any significant growth inhibition of YD-10B cells. Moreover, REV attenuated OSCC cell invasion in a dose-dependent manner (Fig. 6a). Treatment of these cells with REV dramatically abolished RCP-induced Zeb1 expression and MT1-MMP expression (Fig. 6b) as well as OSCC invasiveness (Fig. 6c). REV also efficiently attenuated β1 integrin-induced OSCC invasion (Fig. 6d). Consistent with these findings, REV ablated RCP-induced OSCC invasion compared to vector transfectant when the cells were co-cultured with oral CAFs (Fig. 6e), implying that REV suppresses RCP-induced changes in the tumor microenvironment of OSCC. Furthermore, REV efficiently blocked the RCP-induced growth of OSCC in 3D Matrigel (Fig. 6f, g). Moreover, we noticed that REV intensely blocks RCP-induced β1 integrin expression and filopodia formation at the leading edge of OSCC (Fig. 6h, i). Therefore, these data indicate that REV inhibits RCP-induced salvage of β1 integrin from lysosomal degradation and thus reduces Zeb1 expression, resulting in attenuation of OSCC invasiveness.

## Discussion

Accumulating evidence has suggested the critical role of RCP in endosome recycling and cancer progression. While the majority of studies have pointed out the oncogenic function of RCP, a recent report showed that RCP exerts a tumor suppressive role in mammary tumors through destabilizing ErBB2^15^, indicating the types of cancer- and context-dependent characteristics of RCP in cancer progression. In the present study, we show for the first time that RCP positively regulates OSCC invasiveness through Zeb1 and MT1-MMP expression. Furthermore, we provide evidence that REV efficiently attenuates RCP-induced OSCC invasion through the downregulation of β1 integrin and consequent Zeb1 expression.

RCP plays a prominent role in the invasion and metastasis of various cancers through recycling endosomes and thereby recapitulating integrins and growth factor receptors^8^. Indeed, we recently provide evidence that RCP stabilizes β1 integrin from lysosomal degradation and activation of the EGFR downstream signaling cascade, culminating in aggravation of cancer invasion and metastasis^11^. In the present study, we extended our study to investigate the role of RCP in the invasiveness of one of the aggressive types of HNSCC, OSCC, and uncovered that RCP induces OSCC invasion (Fig. 1a, b). In addition, RCP caused a dramatic increase in filopodia filled with β1 integrin around the plasma membrane (Figs. 3a and 6i), indicating the role of RCP in OSCC invasiveness. Furthermore, we observed enhanced invasiveness in RCP-overexpressing OSCC cells compared to vector-transfected cells when they were cocultured with oral CAFs (Figs. 1e and 6e), suggesting that RCP enables OSCC to communicate with oral CAFs in the tumor microenvironment and promotes OSCC invasion. It might be possible that RCP causes OSCC cells to secrete factors activating nearby oral CAFs to promote OSCC invasion. The underlying mechanism by which RCP connects OSCC cells with oral CAFs to regulate OSCC invasion is under current investigation.

Zeb1 is a well-known EMT-TF associated with tumor progression. In addition to participating in the invasion and metastatic dissemination of cancer cells, Zeb1 is implicated in malignant tumor formation, cancer stemness, and resistance to treatment^28^. Unlike in other types of cancers, the role of Zeb1 in HNSCC and OSCC is now receiving attention. Recently, Zeb1 was identified as an indicator for recurrent OSCC^29^, and blocking Zeb1 resulted in regression of OSCC metastasis^30^. In addition, a higher level of Zeb1 expression was detected in advanced tongue squamous cell carcinoma compared with neighboring normal epithelia^31^. Furthermore, the association of Zeb1 expression with OSCC invasion was identified through RNA-binding protein quaking (QKI)^32^. In the present study, we underscore the important role of Zeb1 in RCP-induced OSCC invasion. Indeed, Zeb1 was sufficient to increase OSCC invasion (Fig. 2f). Conversely, silencing Zeb1 expression attenuated RCP-induced OSCC invasion (Fig. 2c), unveiling the critical role of Zeb1 in OSCC progression and an important target for OSCC patients. In addition, we identified that Zeb1 is located downstream of the β1 integrin/EGFR signaling axis (Fig. 3b, e). Among several proteolytic enzymes implicated in cancer invasion, RCP and Zeb1 increase MT1-MMP, which is critical for RCP-induced OSCC invasion. In support of our results, MT1-MMP is an important proteolytic enzyme for OSCC EMT and invasion^33,34^.

**Fig. 7:**
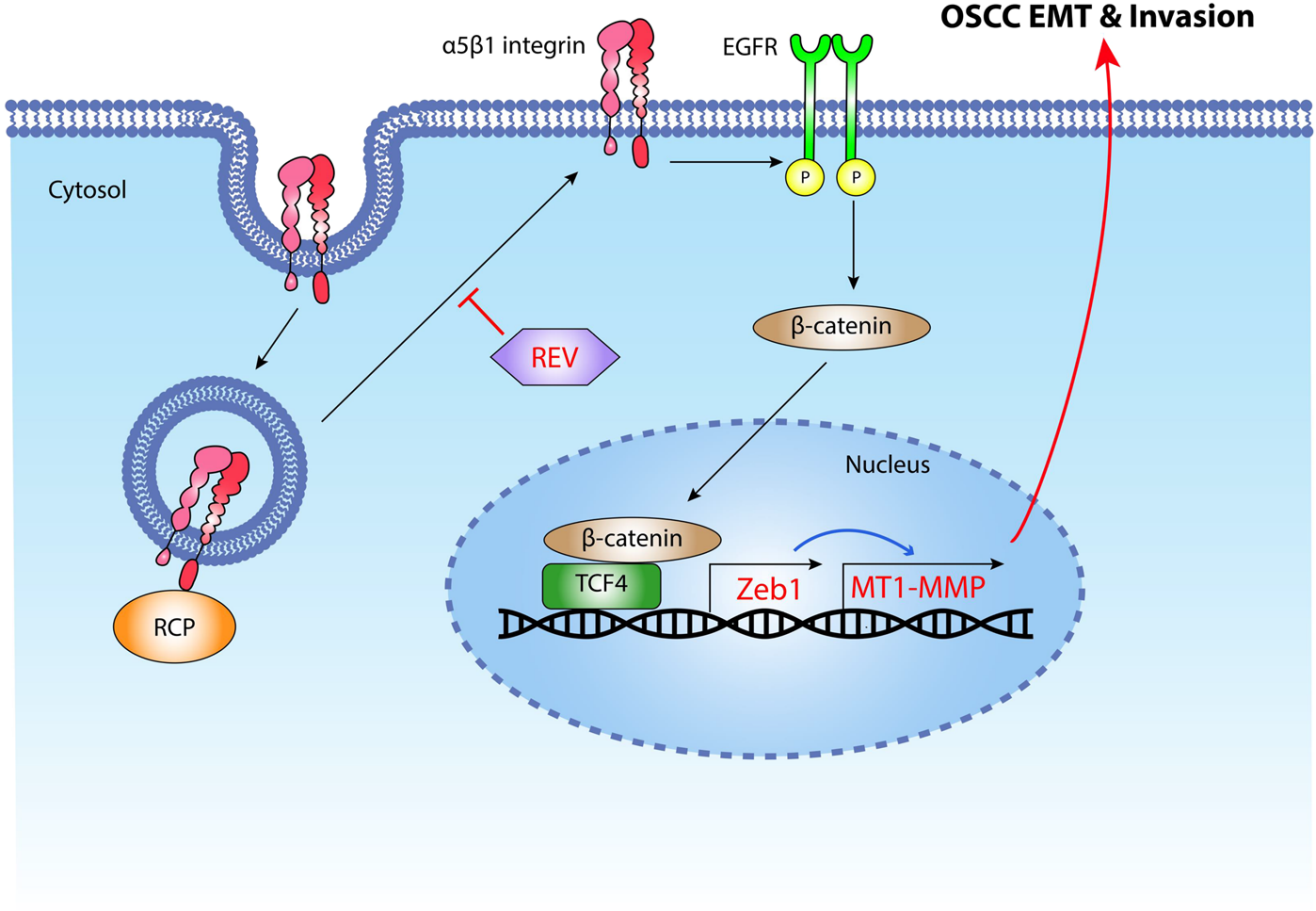
Working model showing how RCP promotes OSCC invasion, which is reversed by REV. RCP induces Zeb1 and MT1-MMP expression through the β1 integrin/EGFR/β-catenin signaling axis to promote OSCC invasion. REV efficiently blocks RCP-induced β1 integrin relocation to the plasma membrane and EGFR activation, resulting in suppression of RCP-induced OSCC invasion.

The naturally occurring polyphenolic compound REV (trans-3,4,5-trihydroxystilbene) has multiple anti-metastatic effects in various cancer cells^27^. REV has been shown to suppress OSCC EMT^35^ and invasion^36^. In this study, we elucidated an additional mechanism by which REV attenuates OSCC progression. We provide evidence that REV blocks the recycling of β1 integrin to the plasma membrane and consequently inactivates EGFR and the downstream signaling cascade for Zeb1 expression (Fig. 6b) and OSCC invasion (Fig. 6c, d). In support of our data, REV has been shown to inhibit Zeb1 expression to reduce the myofibroblast activity of buccal mucosal fibroblasts^37^. In addition, our data show for the first time that REV suppresses RCP-induced OSCC filopodia formation (Fig. 6h, i), which is in good accordance with a previous report showing REV-induced inhibition of filopodia formation in renal cell carcinoma cells^38^. In view of our current results, we present a working model (Fig. 7) showing that RCP promotes OSCC invasion by promoting the recycling of β1 integrin and the activation of the EGFR/β-catenin/Zeb1/MT1-MMP signaling axis and also shows that REV inhibits RCP-induced OSCC invasion.

## Supplementary information

Supplementary information
