# Supplementary material for: Zeb1 for RCP-induced oral cancer cell invasion and its suppression by resveratrol
Source: Exp Mol Med. 2020 Jul 30;52(7):1152–63. doi: 10.1038/s12276-020-0474-1 (PMC8080807; doi:10.1038/s12276-020-0474-1)
Supplement: Supplementary file 1 — Supplementary information [file 12276_2020_474_MOESM1_ESM.docx]

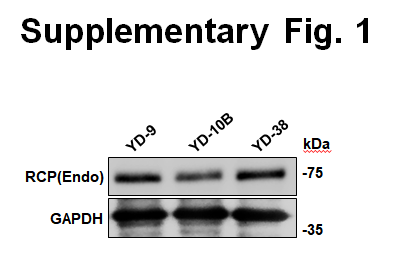


**Supplementary Fig. 1** Immunoblotting of basal RCP expression in OSCC cells.


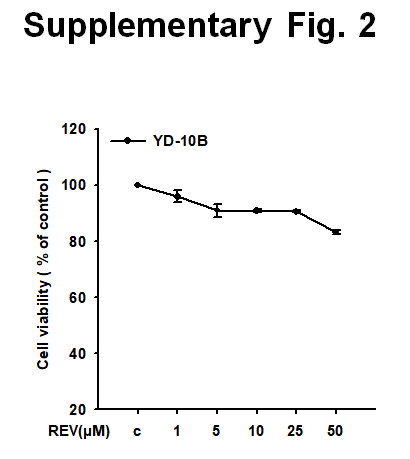


**Supplementary Fig. 2** **The YD-10B cells were treated with indicated amounts of REV for 24 h. MTT assay was performed.**
